# Design, Synthesis and Antitubercular Evaluation of Novel Series of Pyrazinecarboxamide Metal Complexes

**Published:** 2018

**Authors:** Mohsin Ali, Mansoor Ahmed, Saleem Hafiz, Mustafa Kamal, Majid Mumtaz, Seyed Abdulmajid Ayatollahi

**Affiliations:** a *Department of Chemistry, University of Karachi, Karachi-75270, Pakistan. *; b *Department of Pharmaceutical Chemistry, University of Karachi, Karachi-75270, Pakistan. *; c *Department of Microbiology, Sindh Institute of Urology and Transplantation (SIUT), Karachi, Pakistan.*; d *Department of Biotechnology, University of Karachi, Karachi-75270, Pakistan. *; e *Phytochemistry Research Center and Department of Pharmacognosy, School of Pharmacy, Shahid Beheshti University of Medical Sciences, Tehran, Iran.*

**Keywords:** Pyrazinamide, Metal complexes, MGIT 960, ZN method, *Mycobacterium tuberculosis*

## Abstract

The interest in the synthesis of metallic complexes of different drugs to make them more efficient in biological environment of the human body is seen for the last few decades. Wide range of metal complexes are already used in clinical practice which encourages additional research for innovating new metal based drugs, such as metal-mediated antibiotics, anti-parasitic, antiviral, antibacterial, and anticancer compounds. Tuberculosis has been known as one of the most disastrous disease putting burden on health system worldwide. Though the therapeutic agents to combat the disease are well practiced, emergence of multi drug resistant strains makes the treatment strategies more difficult. The following work aims to synthesize copper, ferrous, ferric, cobalt and manganese complexes of renowned anti tuberculosis drug, pyrazinamide (PZ). These compounds were tested for anti-tuberculosis using five multidrug resistant strain of *Mycobacterium tuberculosis*. For this purpose BACTEC MGIT 960 system was used. The drug PZ was also screened with the synthesized complexes. The two complexes of cobalt and manganese proved potent among all of the compounds tested.

## Introduction

Tuberculosis, a disease of antiquity, commonly known as ‘*TB*’ and ‘*white plaque*’, is caused by infection with members of the MTB complex, including *Mycobacterium tuberculosis* itself, Mycobacterium Africanum, Mycobacterium Microti, Mycobacterium Caprae, Mycobacterium Pinnipedii, Mycobacterium Bovis, and Mycobacterium Canettii (1). In 1882, Robert Koch, the Noble prize winner for his discovery, isolated the bacteria, MTB (2). Till mid of 19^th^ century TB was responsible for about a quarter of all deaths in Europe (3). The disease has under gone a resurgence since 1980s owing to the increasingly densely populated urbanization in the developing countries and the increased mobility of human populations (4). Nearly one third of the world’s population has been infected by these bacterial strains during the past few decades rendering it to one of the deadliest diseases globally (5) with the annual incidence rate being 1% of world population (6). Being a contagious disease, it is a major threat for public health globally especially in the developing and under developed densely populated countries (7).

Although the currently practiced pharmacological methods of treatment are very effective against TB, the treatment involves administration of multi-drug regimen over a long period of time, leading to patient noncompliance (8). Furthermore, the emergence of multi-drug resistant TB (Isoniazid and Rifampin) (MDR-TB), extensively drug resistant (XDR-TB) strains and the high prevalence rate of HIV-1 worldwide, are some of the factors which seem to worsen the situation in the future. These factors have highlighted the need for improving the anti-TB drugs by making them more effective thereby increasing the patient compliance (9, 10). 

Pyrazinamide (PZ), a member of the pyrazine family having the general chemical formula C_5_ H_5_ N_3_O is known as a very effective antimycobacterial agent being used in both primary and secondary line treatment schemes. The emergence of strain resistant to PZ represents an important public health problem, as this drug is capable of shortening the tuberculosis therapy from 9–12 months to a period of 6 months (11).

The research has explored that within the cells, metal complexes can participate in reactions that would otherwise be impossible with conventional organic substances only. Though the modern chemotherapy has progressed considerably, there still remains a need for innovative anti-TB agents capable of combating drug resistance by Mycobacterium TB. Complexes of PZ have been reported in literature for their anti-mycobacterial properties (12-18).

Research papers on infectious diseases have demonstrated that serum copper level increases in the patients suffering from tuberculosis. Furthermore, the Cu/Zn ratio signiﬁcantly decreases after a few months of antitubercular therapy with isoniazid, rifampicin, ethambutol, and pyrazinamide, as compared to the ratio at the beginning of the therapy. This could suggest the possible interaction of these drugs with these metal ions resulting in their serum level changes (19-21).

In this research contribution, we explore the synthesis of copper, cobalt, ferric, ferrous, and manganese complexes of PZ which is a very effective antimycobacterial agent and have vast field of applications in primary and secondary line treatment schemes. Besides, this drug is capable of considerably reducing the treatment time of the tuberculosis therapy. Our results demonstrate the successful synthesis of Pyrazinamide-metal complexes using an efficient and relatively easy solution based route. The antitubercular properties of the complexes were thoroughly explored using five resistant species of *M. tuberculosis*. Two metal complexes of PZ exhibit promising results and five strains used in this study were found prone against these complexes over a period of six weeks. 

## Experimental

*Preparation of complexes (PZ)*

The solution of PZ (0.1 M) 20.0 mL in methanol was added, under stirring, into a round bottom flask, containing 10 mL of 0.1 M solution of copper chloride. The resulting mixture was kept stirring for few min at room temperature followed by refluxing on a water bath at 80 ºC for 3 to 4 h. Thus, the solid material formed was filtered using Whatman filter paper and washed by hot methanol to furnish Cu complex of PZ. The same procedure was adopted for the synthesis of complexes of metal (II). 

*Anti-Mycobacterial activity*

Five reference multidrug resistant (*i.e.*, resistant to isoniazid, pyrazinamide, ethambutol and rifampin) strains were used in this study for PZ susceptibility testing using the MGIT 960 system according to the manufacturerꞌs instructions (22), and WHO (23). Since the BACTEC MGIT 960 system is routinely used in laboratory for primary isolation, 500 µL from a positive MGIT tube was sub cultured in vials for susceptibility testing. The drug solutions were prepared by reconstitution of the provided lyophilized drugs with distilled water. The tests with the automated BACTEC MGIT 960 instrumentation were performed with MGIT cultures. 

Each 7.0 mL MGIT tubes were supplemented with 0.8 mL of growth supplement OADS (25). The lyophilized drugs were rehydrated in accordance with the recommended procedure; 100 µL of antibiotic solution (the recommended critical concentration of PZ was 100 μg/mL) was added to a labeled MGIT tube for each drug. 

For the drug-free control, the culture was diluted 1:100 in distilled water before addition to the control tube. The tubes were placed in the proper MGIT rack in a fixed sequence; the rack was incubated in the cabinet drawer and left there until the conclusion of the test was signaled by the instrument. A single concentration of drug (*i.e.*, critical concentration) was tested. 

After bar code scanning all the inoculated tubes were loaded in the instrument and incubated at a temperature of 37 °C. An un-inoculated tube was used as negative control, and incubated for six weeks (42 days).

**Table 1:** Sensitivity of PZ and its complexes against *M. tuberculosis* strains.

**Strains** **(starting date)**	**PZ**	**Fe(II)-PZ**	**Fe(III)-PZ**	**Cu(II)-PZ**	**Co(II)-PZ**	**Ni(II)-PZ**	**Control**
1195 (20-6-16)	Positive (4-7-16)	Positive (4-7-16)	Positive (24-6-16)	Positive (24-6-16)	Negative (2-8-16)	Negative (2-8-16)	Positive (27-6-16)
3029 (20-6-16)	Positive (4-7-16)	Positive (4-7-16)	Positive (27-6-16)	Positive (27-6-16)	Negative (2-8-16)	Positive (18-7-16)	Positive (27-6-16)
1289 (20-6-16)	Positive (4-7-16)	Positive (4-7-16)	Positive (27-6-16)	Positive (27-6-16)	Negative (2-8-16)	Positive (18-7-16)	Positive (27-6-16)
1375 (20-6-16)	Positive (4-7-16)	Positive (24-6-16)	Positive (24-6-16)	Positive (24-6-16)	Negative (2-8-16)	Positive (18-7-16)	Positive (27-6-16)
2029 (20-6-16)	Positive (4-7-16)	Positive (27-6-16)	Positive (27-6-16)	Positive (27-6-16)	Negative (2-8-16)	Negative (2-8-16)	Positive (27-6-16)

**Figure 1:**
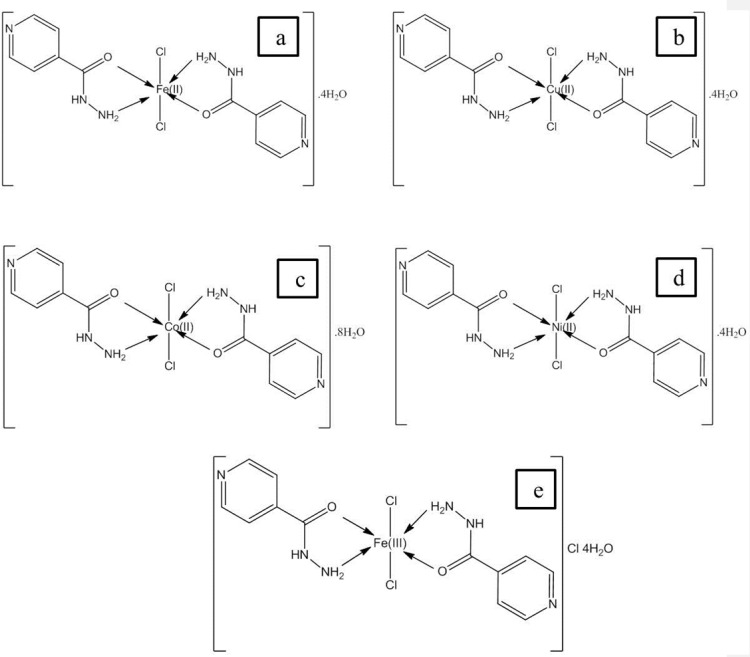
Structure of Metal complexes of PZ (a) Cu(II), (b) Mn(II), (c) Co(II), (d) Fe(II) and (e) Fe(III).

**Figure 2:**
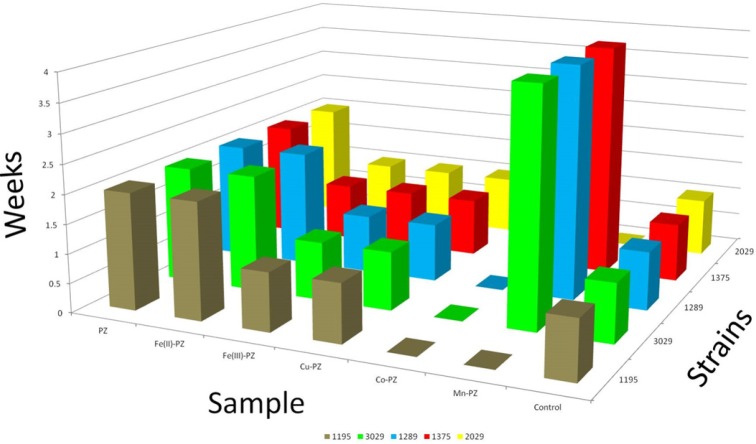
Graphical representation of Mycobacterium with respect to time

The MIGIT 960 System flags the completion of a DST when the growth unit (GU) of the growth control reaches 400 and reports S for susceptible or R for resistant, as well as a GU value for each drug containing MGIT tube on the printout. An isolate was interpreted to be susceptible when the GU of a drug containing MGIT tube was equal to or less than 100 or as resistant when the GU was greater than 100.

If an isolate was interpreted to be resistant, a smear was made and stained by ZN method to prove the presence of AFB with morphology compatible with that of *M. tuberculosis* and the absence of contaminants.

## Results

Structural characterization of complexes will be reported elsewhere and proposed structures are shown in Figure 1. In all complexes, metal to drug ratio is 1:2, whereas charge of metal ions is neutralized by two chloride ions that is present in coordination sphere. It is noticeable in structure (e) the oxidation state of iron is 3+ thus one chloride ion is also present with the water molecules.

*Anti-Mycobacterial activity*

PZ was used as reference drug against five resistant strains of *M. tuberculosis*. This study was carried out for a period of six weeks. The growth was monitored in order to check the resistance of these strains against the synthesized metal complexes derived from PZ. Co (II)-PZ showed significant resistant and found most potent against all strains over the period of six weeks. Ni (II)-PZ also proved resistant over the period of six weeks but against only two strains (1195 and 2029). It also showed resistance against the remaining three strains for a period of four weeks. The details of five complexes with their activity are given in Table 1 and the results are further elaborated in Figure 2.

## Discussion

It is reported that one third of human population is latently infected with MTB, with loss of millions of lives every year. With emergence of MDR-TB and later on XDR-TB there is an immense need to discover and to develop new drugs or to modify the existing drugs to overcome the wide spread dissemination of drug resistant strains of *M. tuberculosis. *TB control program will therefore require finding drugs that are effective against the prevalent drug resistant tubercle bacilli by screening of possible compounds that would be active against MTB in any community. For this reason we have prepared the iron (II), iron (III), copper (II), cobalt and manganese (II) complexes of PZ to test for its anti-tuberculosis activity. *In-vitro* anti-tuberculosis activities of these compounds were checked by BACTEC MGIT 960 (M960). Metal-PZ susceptibility testing using the MGIT 960 system was performed according to the manufacturerꞌs instructions (Becton, Dickinson and Company, Sparks, MD) (22).

Pyrazinamide (PZ) is one of the first-line drugs that are being currently used for the treatment of both drug-resistant as well as for drug-susceptible tuberculosis (TB). In order to be effective against *M. tuberculosis, *PZ (prodrug) needs to be converted into its active moiety, pyrazinoic acid, by the mycobacterial enzyme pyrazinamidase (PZase) (24-26). In non MDR-TB, the duration of treatment will be shortening to 6 months instead of 9 months by the addition of PZ to rifampin and isoniazid (27, 28). 

Loss of PZase activity is a common finding (29, 30). Mutations in pncA, the gene coding for PZase, are now considered as the major cause of resistance in PZ-resistant clinical isolates (31-33). It is reported that not all PZ-resistant MTB isolates have mutations in the pncA gene, but another gene (rpsA) is indicated to be involved in low-level PZ resistance (34, 35).

Kurbatova *et al.* (36) reported that there was an increasing PZA resistant annually in United States from 2.0% to 3.3% during 1999–2009, among MTBC cases: 2.2% of non-MDR cases and among multidrug-resistant (MDR) cases, 38.0% were found to be PZ-resistant. In contrast, PZA resistance was reported in 1.0–1.2% of all tuberculosis cases in Australia during 2008–2009 (37). 

Hasan *et al.* (2012) reported pyrazinamide resistance in 7.5% of rifampicin susceptible cases of MTB in Pakistan. Decreased odds of successful clinical outcomes are also reported in pyrazinamide resistant as compared with pan-susceptible TB (38). Whereas, Ghafoor *et al.* (2012) indicated that resistance to Pyrazinamide was found to be 64.6% in MDR-MTB cases in KPK province of Pakistan.

Another study conducted by Hasan *et al.* (2009) indicated a steady increase in resistance among MTB isolates and the emergence of XDR strains of MTB in Karachi, Pakistan during 1990-2007. They reported that between 1990 and 2007 there was an overall increasing trend of resistance against all first-line drugs, including MDR. It was also observed that PZA resistant was increased 20-40% during year 2000-2007. The observations of an increasing level of resistance in MTB and in particular the evolution of XDR strains is a serious issue particularly in a low-resource setting such as Pakistan (38).

Regarding PZA resistant from other part of the globe, in South Korea drug resistance surveys conducted between 1999-2004 demonstrated a significant increase of PZA resistance in new cases from 0.8-2.1% (39). Similar study reported PZA resistance varied from 3.5-15.9% with no clear trend in retreatment cases (39). Similarly, PZA resistance in 6% of non-MDR cases and 49% of MDR tuberculosis isolates has been reported from Thailand (40). The proportion of MDR tuberculosis cases with PZA resistance ranged from 36-85% in other reports (41-45). It is obvious from above studies that since PZA is an essential part of anti tuberculus treatment the increasing trend in PZA resistance makes it an important public health problem. Factors associated with PZA resistance among cases of *M. tuberculosis *said to be less well understood, suggesting that bacterial lineage, rather than host characteristic, was the primary association (46-51). 

In our study the highest activity was displayed by the Co and Mn derivatives compounds of pyrazinamide drug. Thus, the presence of Co and Mn at carbonyl and amide group on the pyrazine seems favorable for antibacterial activity against

Further studies are underway aimed at the detailed elucidation of structure–activity relationships in the class of PZA metal complex to discover a suitable drug candidate.
